# Collagen-induced arthritis in C57BL/6 mice is associated with a robust and sustained T-cell response to type II collagen

**DOI:** 10.1186/ar2319

**Published:** 2007-10-29

**Authors:** Julia J Inglis, Gabriel Criado, Mino Medghalchi, Melanie Andrews, Ann Sandison, Marc Feldmann, Richard O Williams

**Affiliations:** 1 Kennedy Institute of Rheumatology, Imperial College London, 1 Aspenlea Road, London W6 8LH, UK; 2 Department of Histopathology, Imperial College London, Charing Cross Hospital, Fulham Palace Road, London W6 8RF, UK

## Abstract

Many genetically modified mouse strains are now available on a C57BL/6 (H-2^b^) background, a strain that is relatively resistant to collagen-induced arthritis. To facilitate the molecular understanding of autoimmune arthritis, we characterised the induction of arthritis in C57BL/6 mice and then validated the disease as a relevant pre-clinical model for rheumatoid arthritis.

C57BL/6 mice were immunised with type II collagen using different protocols, and arthritis incidence, severity, and response to commonly used anti-arthritic drugs were assessed and compared with DBA/1 mice. We confirmed that C57BL/6 mice are susceptible to arthritis induced by immunisation with chicken type II collagen and develop strong and sustained T-cell responses to type II collagen. Arthritis was milder in C57BL/6 mice than DBA/1 mice and more closely resembled rheumatoid arthritis in its response to therapeutic intervention. Our findings show that C57BL/6 mice are susceptible to collagen-induced arthritis, providing a valuable model for assessing the role of specific genes involved in the induction and/or maintenance of arthritis and for evaluating the efficacy of novel drugs, particularly those targeted at T cells.

## Introduction

Rheumatoid arthritis (RA) is a highly inflammatory chronic polyarthritis that causes joint destruction, deformity, and loss of function. Sequelae include pain, disability, and increased mortality. A role for CD4^+ ^T cells in the pathogenesis of RA is inferred from the strong HLA-DR association as well as the large numbers of major histocompatability complex class II-positive cells found in close proximity to activated CD4^+ ^T cells in inflamed joints. Furthermore, immunisation of transgenic mice expressing RA-associated HLA-DR4/DR1 haplotypes with type II collagen results in arthritis [1,2] and reveals a single immunodominant epitope (amino acids 261 to 273) that overlaps the immunodominant epitope in DBA/1 mice with collagen-induced arthritis (CIA) (256 to 270) [1,3].

The identification of tumour necrosis factor-alpha (TNF-α) as a key mediator of inflammation in RA has led to the development of TNF-α-blocking biologics that control disease activity, but there remains a need for therapies capable of modulating the underlying immune response [4].

Pre-clinical assessment of therapeutics for RA has relied largely on murine models of arthritis, particularly the CIA model, in which mice are immunised with heterologous type II collagen in complete Freund's adjuvant (CFA) [5]. The development of CIA is strain-dependent, with H-2^q ^and H-2^r ^haplotypes showing the greatest degree of susceptibility. The DBA/1 strain (H-2^q^) is the most commonly used strain for pre-clinical testing of potential anti-arthritic drugs and was successfully used to predict the beneficial effects of TNF-α blockade [6,7]. However, although CIA in DBA/1 mice has been extremely useful for testing drugs with anti-inflammatory properties, its usefulness for assessing T cell-targeted therapies is limited to some extent by the relatively acute nature of the disease.

A further limitation of the classic CIA model in DBA/1 mice is that most transgenic and knockout strains of mice are on a C57BL/6 (B6) background (H-2^b^), which is regarded to be relatively resistant to arthritis induction when bovine type II collagen is used as an immunogen [8,9]. To circumvent this problem, genetically modified strains have generally been backcrossed for a minimum of eight generations onto the DBA/1 background, which introduces a delay of 1 to 2 years. However, it has been reported that, contrary to previous findings, B6 mice are indeed susceptible to arthritis induced by chicken type II collagen [10-12], although many groups have been unable to induce arthritis in this strain in a reproducible manner [8].

The primary aims of this project were to characterise CIA in the C57BL/6 mouse clinically and histologically and to analyse cellular and humoral immune responses to type II collagen during the course of the disease. We show that B6 mice develop a chronic form of CIA and that this model closely resembles human RA in terms of its disease course, histological findings, and in its response to commonly used anti-arthritic drugs. We also show that B6 mice develop a sustained T-cell response to chicken collagen as well as to autologous (mouse) collagen.

## Materials and methods

### Purification of type II collagen

Bovine collagen was purified from articular cartilage, and mouse and chicken collagens were purified from non-articular (sternal) cartilage. All collagens were prepared by pepsin digestion and salt fractionation according to established procedures [13]. Lathyritic rat type II collagen (a gift from Lars Klareskog, formerly of Uppsala, Sweden) was prepared without pepsin.

### Induction and assessment of arthritis

Ten- to 12-week-old male mice were used for all procedures, were housed in groups of 10, and were maintained at 21°C ± 2°C on a 12-hour light/dark cycle with food and water *ad libitum*. All experimental procedures were approved by the local ethical review process committee and the UK Home Office. DBA/1 mice were bred at the Kennedy Institute of Rheumatology (London, UK) and B6 mice were purchased from Harlan UK (Bicester, Oxfordshire, UK). To reduce the risk of fighting amongst males, mice from different cages were not mixed beyond 6 weeks of age. All mice were immunised intradermally in two sites at the base of the tail with 200 μg of bovine, chicken, or mouse type II collagen in CFA as described previously [13]. To prepare the CFA, 100 mg of desiccated killed *Mycobacterium tuberculosis *H37Ra (BD Biosciences, Oxford, Oxfordshire, UK) was ground with a pestle and mortar to produce a fine powder and then suspended in 30 mL of incomplete Freund's adjuvant (BD Biosciences). It was observed that fighting amongst male mice reduced the incidence of arthritis. Hence, to reduce the risk of fighting, mice from different cages were not mixed beyond 6 weeks of age. Each experiment was performed on a minimum of two occasions.

For macroscopic assessment of arthritis, the thickness of each affected hind paw was measured daily with microcalipers (Kroeplin GmbH, Schlüchtern, Germany) and the diameter was expressed as an average for inflamed hind paws per mouse. Animals were also scored for clinical signs of arthritis [13] as follows: 0 = normal, 1 = slight swelling and/or erythema, 2 = pronounced oedematous swelling, and 3 = joint rigidity. Each limb was graded thus, allowing a maximum score of 12 per mouse. After completion of the experiment, mice were sacrificed and hind paws were immersion-fixed in 10% (vol/vol) buffered formalin and decalcified with 5.5% EDTA (ethylenediaminetetraacetic acid) in buffered formalin.

For histological assessment of arthritis, arthritic mice were killed up to 2 weeks after disease onset (early arthritis, *n *= 8) or 6 to 8 weeks following onset (late arthritis, *n *= 8). Joints were decalcified and paraffin-embedded, and sections (10 μm) were stained (haematoxylin and eosin) for conventional histology. Joints were classified according to the presence or absence of inflammatory cell infiltrates (defined as focal accumulations of leukocytes). Histological analysis was performed in a blinded fashion by a trained histopathologist (AS) (*N *= 8 per point).

### Analysis of antibody production

Anti-collagen antibody isotypes were assessed in the serum of mice with early or late arthritis. Enzyme-linked immunosorbent assay (ELISA) plates (Thermo Fisher Scientific, Rochester, NY, USA) were coated with 5 μg/mL of type II collagen dissolved in Tris buffer (0.05 M Tris, containing 0.2 M NaCl, pH 7.4), blocked with 2% bovine serum albumin, and then incubated with serial dilutions of test sera. A standard curve was created for each assay by including serial dilutions of a reference sample on each plate. The reference sample was arbitrarily assigned an antibody concentration of 1 AU/mL. Bound IgG1 or IgG2a/c was detected by incubation with horseradish peroxidase-conjugated sheep anti-mouse IgG1 (BD Biosciences), or an antibody that recognises both IgG2a and IgG2c (BD Biosciences), followed by TMB (3,3', 5,5'-tetramethylbenzidine) substrate. Optical density was measured at 450 nm. Antibody concentrations for each serum sample were obtained by reference to the standard curve (*N *= 8 per point).

### Analysis of T-cell activity

Inguinal lymph nodes were excised from mice with early or late arthritis. Lymph node cells (LNCs) were cultured in RPMI 1640 containing foetal calf serum (10% vol/vol), 2-mercaptoethanol (20 μM), L-glutamine (1% wt/vol), penicillin (100 U/mL), and streptomycin (100 μg/mL) in the presence or absence of type II collagen or the synthetic collagen fragment CII256-270 (both at 50 μg/mL). After 48 hours, 100 μL of culture medium was carefully removed for measurement of cytokines and the remaining cells were pulsed with 1 μCi ^3^H thymidine per well for a further 18 hours. Cells were then harvested and plates were assessed for ^3^H thymidine incorporation. Each assay was performed on a minimum of three occasions. Secreted interferon-gamma (IFN-γ), interleukin (IL)-5, and IL-10 were measured in the culture supernatant by sandwich ELISA using capture and detection antibody pairs (BD Biosciences).

### Drug therapy

The therapeutic responses of arthritic B6 and DBA/1 mice to intraperitoneal administration of dexamethasone (0.5 mg/kg daily), anti-TNF monoclonal antibody (mAb) (TN3-19.12; 300 μg every 3 days), methotrexate (0.75 mg/kg every 3 days), or indomethacin (2.5 mg/kg daily) were assessed. The therapeutic response was defined as the percentage reduction in clinical score following 10 days of therapy relative to mice treated with vehicle alone. Each experiment was performed twice.

### Statistical analysis

Statistical analysis was performed by one-way analysis of variance followed by Dunnett multiple comparisons test, where appropriate.

## Results

### Induction of arthritis in B6 mice

**Figure 1:**
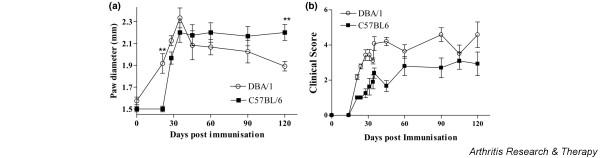
Collagen-induced arthritis (CIA) in B6 and DBA/1 mice. Mice were immunised with type II collagen in complete Freund's adjuvant, and paw diameter **(a) **and clinical score **(b) **were measured for 120 days (*n *= 10 arthritic mice per group). **(a) **Paw swelling reached a peak in DBA/1 mice on day 30 and declined thereafter. In contrast, in B6 mice, paw swelling, although less pronounced, remained elevated up to day 120. **P < 0.01. **(b) **The clinical score was less in B6 CIA than DBA/1 CIA throughout most of the period studied. *P < 0.05.

We first compared bovine, chicken, and mouse collagen type II for their ability to induce arthritis in B6 mice (Table 1). Only chicken collagen was able to induce arthritis in B6 mice, with a maximum incidence of 61.7% and mean day of onset of 29.4 ± 1.3 days after primary immunisation. This is in contrast to DBA/1 mice, in which collagen from all species induced arthritis (Table 1). We then compared the clinical progression of arthritis in B6 mice with our standard CIA in DBA/1 mice, immunised with bovine CII. Hind paw swelling was assessed up to 120 days after immunisation. Paw swelling in B6 mice was significantly less than in DBA/1 mice on day 21 after immunisation but was significantly greater on day 120 after immunisation (Figure 1a). However, clinical scores of DBA/1 mice with CIA were higher than those of B6 mice, indicating that arthritis was milder in B6 mice than DBA/1 mice (Figure 1b).

**Table 1:** Incidence, mean day of onset, and maximum clinical score of B6 and DBA/1 mice with collagen-induced arthritis

	Species of CII used for immunisation					
	
	Bovine		Chicken		Mouse	
	DBA/1	C57BL/6	DBA/1	C57BL/6	DBA/1	C57BL/6

Incidence	94	0	96	61	30	0
Mean day of onset	24.4 ± 1.9	0	18.3 ± 2.5	29.4 ± 1.3	81.7 ± 11.5	0
Maximum clinical score	4.3 ± 0.9	0	5.8 ± 2.3	3.1 ± 2.2	2.6 ± 1.0	0

There were 20 or more mice per group (data pooled from three independent experiments).

**Figure 2:**
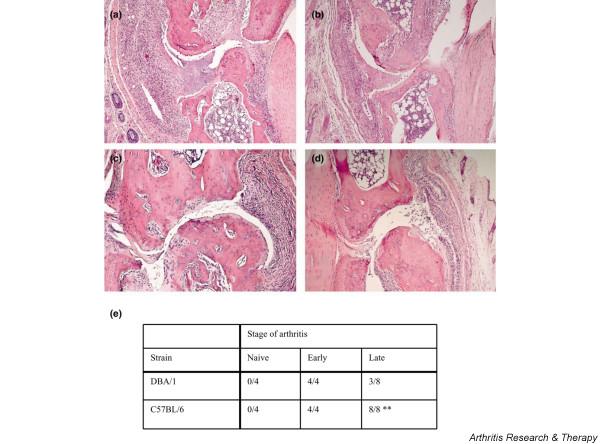
Chronic inflammatory infiltrate in B6 mice with collagen-induced arthritis. Histological assessment of arthritis was carried out in early arthritis (up to 2 weeks after onset, *n *= 8) and late arthritis (6 to 8 weeks after onset, *n *= 8). **(a) **Severe joint destruction with massive accumulation of polymorphonuclear cells (PMNs) was observed in DBA/1 in early arthritis. **(b) **In B6 mice, the infiltrating cells were predominantly mononuclear in early arthritis and there was less joint erosion. **(c) **In late arthritis, the inflammatory response largely resolved in DBA/1 mice, although the joint destruction was not reversed. **(d) **The inflammatory response remained active in B6 mice in late arthritis and there was progressive joint erosion. Original magnification, × 100. **(e) **The numbers of joints showing foci of inflammatory cells (lymphocytes and PMNs) in the joint were compared in early and late arthritis. **P < 0.01.

To assess the histological outcome in the two models, hind paws were fixed, sectioned, and stained with haematoxylin and eosin. In the early stages of CIA, inflammatory infiltrates were found in both the DBA/1 (Figure 2a) and B6 (Figure 2b) joints. However, at late stages of disease, only 37.5% of DBA/1 mice studied had inflammatory infiltrates (Figure 2c,e). In contrast, 100% of B6 joints studied had inflammatory infiltrates in both early and late arthritis (Figure 2d,f).

### Comparison of anti-collagen IgG profiles in B6 and DBA/1 mice

**Figure 3:**
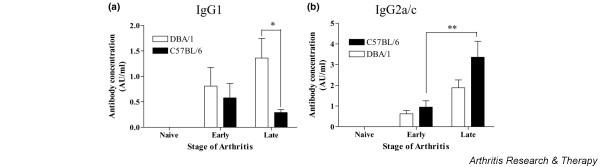
Comparison of anti-collagen IgG isotypes in DBA/1 and B6 mice with collagen-induced arthritis. Serum from naïve and arthritic mice were analysed for anti-collagen antibodies. IgG1 **(a) **and IgG2a/c **(b) **anti-collagen isotypes were quantified in naïve mice and mice with early (up to 2 weeks after onset) and late (6 to 8 weeks after onset) arthritis after immunisation (*n *= 8). *P < 0.05, **P < 0.01.

Circulating anti-collagen IgG1 and IgG2a/c isotypes were assessed by ELISA (Figure 3). At early stages of disease (up to 2 weeks after onset), the two strains of mice had similar levels of collagen-specific IgG1 (Figure 3a) and IgG2a/c (Figure 3b). In late disease (6 to 8 weeks after onset), titres of both IgG1 and IgG2a/c had increased modestly in DBA/1 mice. In contrast, in late stages of disease, levels of IgG1 had fallen, whereas levels of IgG2a/c had risen dramatically in B6 mice, indicating a predominant Th1 immune response.

### T-cell responses in B6 and DBA/1 mice

**Figure 4:**
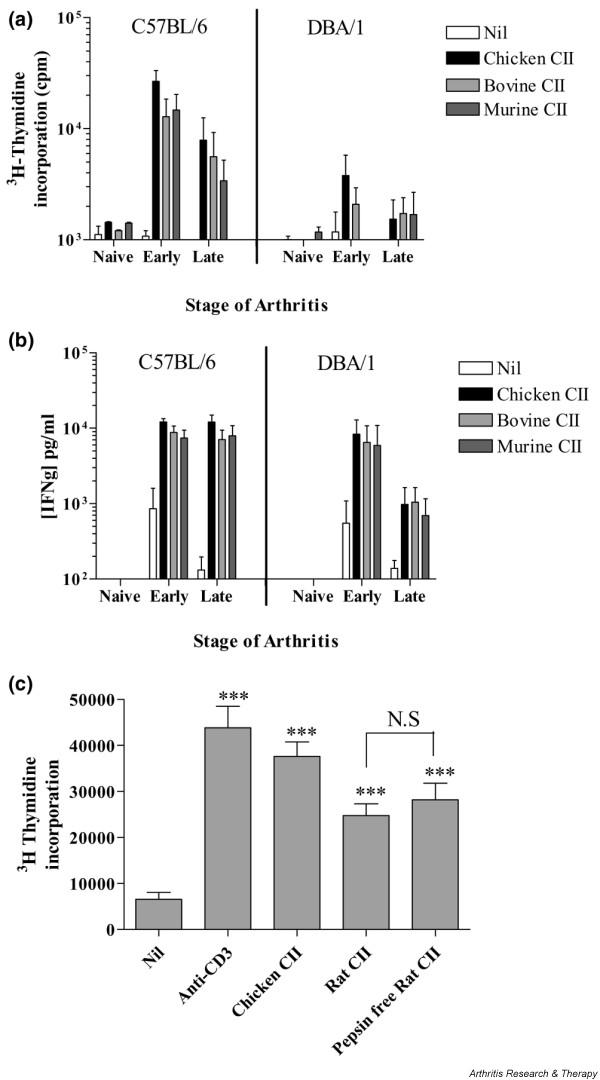
Sustained T-cell responses to collagen type II in B6 mice with collagen-induced arthritis. Inguinal lymph node cells were cultured from naïve mice or mice with early (up to 2 weeks after onset) or late (6 to 8 weeks after onset) arthritis (*n *= 8). Cells were cultured for 72 hours with bovine, chicken, and murine collagen type II. **(a) **Proliferation was assessed by ^3^H thymidine incorporation. **(b) **Interferon-gamma (IFN-γ) secretion was assessed in the culture supernatant by enzyme-linked immunosorbent assay. **(c) **Proliferative responses of T cells from collagen-immunised B6 mice to type II collagen purified with or without pepsin were compared. Responses to chicken collagen and anti-CD3 monoclonal antibody were also measured. Proliferation was assessed by ^3^H thymidine incorporation. ***P < 0.001. N.S., not significant.

To further investigate the T-cell responses in the different strains, LNCs were isolated from DBA/1 and B6 mice before immunisation, up to 2 weeks after disease onset (early arthritis), or 6 to 8 weeks after onset (late arthritis). Assessment of proliferation and IFN-γ production in response to collagen of different species *in vitro *revealed that LNCs from B6 and DBA/1 mice with either early or late arthritis responded to chicken, bovine, and mouse CII (Figure 4a,b). Of particular note were the strong proliferative and cytokine responses to autologous (mouse) collagen in the LNC cultures from B6 mice, providing evidence of autoimmunity at the T-cell level. However, LNCs from arthritic B6 mice failed to respond to the collagen peptide CII256-270 (which represents the immunodominant epitope recognised by T cells from DBA/1 mice in the context of I-Aq), whereas LNCs from DBA/1 mice were responsive (data not shown). This indicates differences in the T-cell epitope specificities between the two strains. IL-5 and IL-10 were not detected in the cultures (data not shown).

It has been reported that pepsin contamination contributes to the high levels of T-cell reactivity observed in some strains of mouse and rat immunised with pepsin-digested collagen [14]. To assess the contribution of pepsin to the anti-collagen T-cell response in B6 mice, we compared the responses of LNCs from arthritic B6 mice to lathyritic pepsin-free rat collagen and to pepsin-digested rat collagen [15] (Figure 4c). Proliferative responses to rat collagen were similar irrespective of whether pepsin was used for digestion. We therefore concluded that the T cells from B6 mice were responding specifically to collagen and not to contaminating pepsin.

### Validation of the B6 model for therapeutic studies

**Figure 5:**
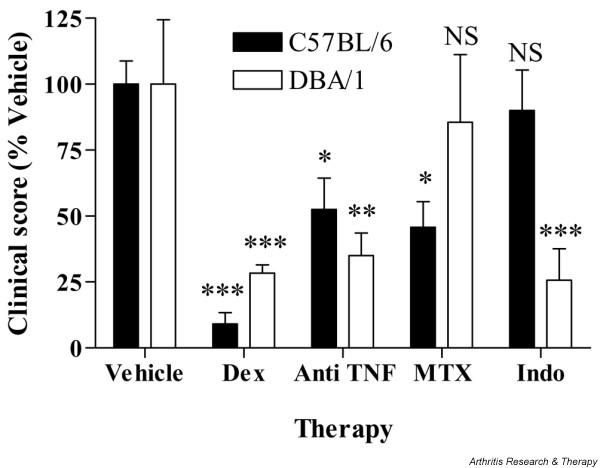
Collagen-induced arthritis in B6 mice is a valid model for testing anti-arthritic compounds. Arthritic B6 or DBA/1 mice were treated from the time of onset of arthritis with dexamethasone (Dex) (0.5 mg/kg per day), anti-tumour necrosis factor (TNF) monoclonal antibody (300 μg every 3 days), methotrexate (MTX) (0.75 mg/kg every 3 days), or indomethacin (Indo) (2.5 mg/kg daily) or the relevant vehicle. After 10 days of therapy, the clinical score was assessed and expressed as a percentage of vehicle-treated mice (*n *= 8 per group). Experiments were repeated twice. Data shown are from one representative study. *P < 0.05, **P < 0.01, ***P < 0.001. NS, not significant.

We next assessed the therapeutic profile of arthritic B6 mice to drugs commonly used to treat RA, including a corticosteroid (dexamethasone), a TNF-blocking biologic (anti-TNF mAb), a disease-modifying anti-rheumatic drug (methotrexate), and a nonsteroidal anti-inflammatory drug (indomethacin). Clinical score was assessed, as a measure of spread of disease progression. As expected, dexamethasone and anti-TNF mAb gave clear reductions in clinical score of at least 75% and 50%, respectively, following 10 days of therapy in both DBA/1 and B6 mice with CIA (Figure 5). In contrast, whereas methotrexate reduced clinical score in arthritic B6 mice by 50%, no significant effect on disease severity was observed in arthritic DBA/1 mice (Figure 5). Likewise, as previously reported, indomethacin reduced clinical score in the DBA/1 mouse by more than 50% but had no significant effect in B6 mice, indicating that it does not alter progression of the disease in this model, as occurs in human RA.

## Discussion

The model of CIA, a T cell- and cytokine-dependent disease, in DBA/1 mice has led to increased understanding of RA and has facilitated the development of novel biologics, such as TNF-blocking therapies [16]. However, the apparent resistance of strains normally used to carry modified genes has impeded our ability to rapidly ask basic questions about disease pathogenesis, as a 1- to 2-year backcross to DBA/1 mice is needed. Our aim was to comprehensively assess the susceptibility of B6 mice to CIA and compare the disease with the 'classic' model in DBA/1 mice.

Our studies showed that chicken, and not bovine, CII was capable of inducing disease in B6 mice, with an incidence of 50% to 75%, an incidence similar to that previously described [10-12]. This is in contrast to DBA/1 mice, in which bovine, mouse, and chicken CII all induced disease, with an incidence of 80% to 100%. This may account for reports of resistance to CIA in B6 mice, in which bovine CII was used for immunisation [8]. Other confounding factors could include the quality of collagen preparation, or substrains of B6 mice, and it is important to note that our study was carried out with B6 mice purchased from Harlan UK, although we have obtained similar results with B6 mice from Charles River UK Ltd. (Margate, Kent, UK).

The phenotype of arthritis was milder in B6 mice than in DBA/1 mice, with less swelling and a more gradual increase in clinical score. Histological assessment of the hind paws from arthritic DBA/1 and B6 mice revealed that, in early arthritis (up to 2 weeks after onset), there was a similar degree of inflammatory cell infiltration in the two strains. In contrast, in late arthritis (6 to 8 weeks after onset), inflammatory cell infiltration was reduced in DBA/1 mice compared with B6 mice, although it remains to be established which cell types are present in the joints of B6 CIA.

Assessment of lymph node responses showed that in the B6 mouse, both early and late after immunisation, proliferation and IFN-γ production in response to collagen occurred. Also of note, a strong response was observed in B6 mice to mouse collagen, suggesting the autoimmune nature of the model. It must be noted that bovine and murine collagen did not induce arthritis in B6 mice.

The reason why chicken, and not mouse or bovine, CII is arthritogenic in B6 mice is presumably due to recognition by B6 T cells of a peptide of chicken CII in the context of H-2^b ^class II molecules. This suggests that differences in the amino acid sequence between chicken and mouse/bovine CII are required to break tolerance and induce arthritis. It is intriguing that the T-cell response was greater and more sustained in B6 mice compared with DBA/1 mice, but the reasons for this are unknown. The number and activity of CD4^+^CD25^+ ^regulatory T cells were found to be similar in the two strains (G. Criado, M. Medghalchi, R.O. Williams, unpublished observations). Therefore, we cannot attribute sustained T-cell responses to any obvious defect in regulatory T cells in B6 mice.

It has been proposed that pepsin (used to purify collagen) plays an important role in breaking T-cell tolerance to collagen and that much of the T-cell response in some strains of mice and rat is directed against pepsin or pepsin-modified epitopes of collagen [14]. By showing equivalent responses to CII prepared with and without pepsin using lathyritic collagen [14], we have shown that the T-cell response is not dependent on pepsin in this model, in contrast to rat strains, in which T-cell responses have been shown to be directed mainly against contaminating pepsin [15]. However, we cannot exclude the possibility that pepsin contributes to the breaking of tolerance during immunisation, and we were unable to obtain lathyritic chicken type II collagen in order to test this hypothesis. However, the mycobacterial component of CFA provides many factors that are able to break tolerance via activation of Toll-like receptors.

The therapeutic profile of CIA in the B6 mouse was similar to that of RA, with a therapeutic action of methotrexate at a dose comparable to human therapy. This is in contrast to CIA in DBA/1 mice, in which methotrexate had no effect. One of the anti-inflammatory mechanisms of methotrexate is thought to be due to increased adenosine production [17]. Adenosine acts via G-protein-coupled receptors to increase cAMP levels, which is known to reduce inflammation [18]. It was recently reported that DBA/1 mice, but not B6 mice, are genetically resistant to the effects of methotrexate, due to defective adenosine accumulation [19]. This is of particular significance as methotrexate is now regarded as the 'gold standard' small-molecular-weight drug for RA and is frequently used in combination with biologics, such as anti-TNF therapy [20]. There is, therefore, an increasing need to model the anti-arthritic effects of methotrexate in combination with other therapies in order to optimise treatment regimens and to identify possible interactions. Likewise, indomethacin did not slow the disease progression of CIA in B6 mice, as in RA, but significantly reduced the disease severity of CIA in DBA/1 mice [21].

## Conclusion

In summary, we have confirmed that inflammatory, destructive arthritis can be induced reproducibly in the B6 mouse using chicken type II collagen. The disease in B6 mice is milder, but more chronic, with more pronounced and more persistent T-cell responses. The maintained presence of inflammatory cell infiltrate and the response of the disease in B6 mice to anti-arthritic drugs such as methotrexate show a good correlation with human RA. We therefore propose that this model will be useful for testing new therapeutics, especially directed against T cells, in addition to investigating mechanisms of action of current therapies such as methotrexate.

## Abbreviations

CFA = complete Freund's adjuvant; CIA = collagen-induced arthritis; ELISA = enzyme-linked immunosorbent assay; IFN-γ = interferon-gamma; IL = interleukin; LNC = lymph node cell; mAb = monoclonal antibody; RA = rheumatoid arthritis; TNF-α = tumour necrosis factor-alpha.

## Competing interests

The authors declare that they have no competing interests.

## Authors' contributions

JJI was the main investigator, carried out most of the experiments, and contributed to the preparation of the manuscript. GC carried out some experiments and contributed to the preparation of the manuscript. MM performed some experiments. MA performed cytokine ELISAs. AS analysed joint histology. MF contributed to the preparation of the manuscript. ROW was the principal investigator, designed the study, and contributed to the preparation of the manuscript. All authors read and approved the final manuscript.
